# Dynamic transcriptome sequencing and analysis during early development in the bighead carp (*Hypophthalmichthys nobilis*)

**DOI:** 10.1186/s12864-019-6181-4

**Published:** 2019-10-28

**Authors:** Jianjun Fu, Wenbin Zhu, Lanmei Wang, Mingkun Luo, Feibiao Song, Zaijie Dong

**Affiliations:** 1 Key Laboratory of Freshwater Fisheries and Germplasm Resources Utilization, Ministry of Agriculture and Rural Affairs, Freshwater Fisheries Research Center of Chinese Academy of Fishery Sciences, Wuxi, 214081 China; 2 Wuxi Fisheries College, Nanjing Agricultural University, Wuxi, 214081 China

**Keywords:** *Hypophthalmichthys nobilis*, Embryo development, Transcriptome, Dynamic regulation, MTZ, Hatching

## Abstract

**Background:**

Early development is a key process of the life history of fish. However, the relationship between the transcriptome and the dynamic regulation of early development is still uncharacterized in the bighead carp (*Hypophthalmichthys nobilis*). In the present study, we performed transcriptome analysis of six development stages in *H. nobilis*, aiming to understand the dynamic molecular regulation of early development in this fish.

**Results:**

A total of 76,573 unigenes were assembled from clean sequence reads, with an average length of 1768 base. Among which, 41,742 (54.54%) unigenes were annotated to public protein databases, and an additional 59,014 simple sequence repeat (SSR) loci were identified among the unigenes. Furthermore, 30,199 differentially expressed transcripts (DETs) (fold change > 4 or < 0.25, and the false discovery rate FDR < 0.01) were observed in comparisons between the adjacent developmental stages, and nine expression patterns (profiles) were simulated using series-cluster analysis across six developmental stages. The unigenes expression level markedly increased after the DS1 stage (early blastula), and the numbers of DETs gradually decreased during subsequent development. The largest transcriptomic change (up- or down-regulated) was detected during the period from DS1 to DS2 (6-somite stage), which was enriched for many biological processes and metabolic pathways related to maternal to zygotic transition (MZT). Distinctly protein-protein interaction (PPI) networks were plotted for DETs during the period from DS1 to DS2. The genes (or proteins) from the same pathways were integrated together, and showed with obvious co-regulation patterns. In the series-cluster analysis, a remarkable profile of gene expression (profile_48) was identified that is probably related to the hatching during *H. nobilis* development, and the strict co-expression of a hatching enzyme gene (*hce1*) with 33 other annotated genes was identified from this profile.

**Conclusions:**

The results indicated that strict dynamic regulation occurs during the early development in *H. nobilis*, especially in embryogenesis before hatching. This study provides valuable new information and transcriptomic resources related to *H. nobilis* early development, and for certain events such as MZT and hatching.

## Background

The bighead carp (*Hypophthalmichthys nobilis*) which is native to central and south China, has been cultured for over a thousand years [1]. Currently, this species is a major aquaculture fish, with an annual worldwide production of over three million tons from 2013 to 2015, principally from China [2]. Unfortunately, a serious decline in the fishery resources of this species has been observed in its natural distribution waters in the past few decades, mainly caused by habitat loss or shrinkage, water pollution, and long-time overfishing [3]. To adapt to the huge market demand, artificial propagation is now the major source of seed to culture *H. nobilis* because of its high yield. Although natural seeds are still available in some Chinese rivers, their supply is insufficient. Breeding design and inbreeding control have not been implemented by every hatchery operator, leading to small effective populations. Inbreeding occurred in a proportion of farms, which caused degradation of seed quality, including a decline in growth performance and abnormally high mortality in the embryogenesis period. Thus, there are urgent tasks that need to be addressed when considering responsible aquaculture practices for *H. nobilis*. Significant effort has been made in the genetic improvement of *H. nobilis* to produce characteristics that are suitable for aquaculture [4]. However, almost no selection progress has been acquired via genetic improvement so far, despite trials of cross breeding with silver carp (*H. molitrix*) being carried out [5, 6]. Meanwhile, genetic research into *H. nobilis* has been carried out, involving population genetic diversity [7], linkage genetic mapping and QTL (quantitative trait locus) analysis [8, 9], sex-associated markers identification [10], and transcriptome studies related to growth [11] and nitrite toxicity [12]. Although these studies have provided basic information for genetic resource utilization and improvement of this species, more genomic and transcriptomic information regarding development will facilitate studies of this species’ genetics and breeding.

Early development is a key process of the life history of fish related to embryogenesis, larval development and growth, and is likely to determine the early survival rate associated with physiological and environmental changes. Advances in high-throughput sequencing, have improved our understanding of how transcripts are regulated during early vertebrate development [13]. Many studies have investigated gene expression in the early development of teleost species, related to the basal processes and mechanisms of embryogenesis and larval development [13–18]. Nevertheless, many questions related to the dynamic changes and regulation of early development remain. The early life stages of *H. nobilis* have been well described by line drawings based on live individuals [19], and the developmental rate and behavior of the early life stages in *H. nobilis* have also been analyzed under different temperatures [20]. The decline in the wild resources of *H. nobilis*, is thought to be related to precise requirements for the survival and recruitment of eggs and larvae. However, although the significant genetic information has been obtained from zebrafish (*Danio rerio*) [14–16] and other teleost species [17, 18], gene expression studies of *H. nobilis* have so far focused on the juvenile or sub adult stages [11, 12], and no data is currently available on the transcriptomic changes in the early development of *H. nobilis*. Therefore, the use of functional genomics approaches would contribute to the identification of molecular signatures associated with normal embryogenesis and early-life events in *H. nobilis*, thus providing deeper insights into dynamics of early development in this species.

RNA-Seq (RNA-sequencing) is regarded as an effective method to identify and quantify transcripts [21]. RNA-Seq has been wildly used in many fishes for comparative transcriptome analyses of different development stages [14–18]. To obtain more genetic information on the molecular basis of embryonic and larval development in *H. nobilis*, the present study performed a transcriptomic analysis in the early life stages of this species. Six early development stages were chosen to investigate and compare the transcriptome changes during early development using RNA-Seq. The results of the present study should lead to a deeper understanding of the biological processes and mechanisms of the dynamic changes during the early development of *H. nobilis*.

## Results

### Overview of the RNA-Seq data

Thirty cDNA libraries were constructed and generated paired-end sequence reads using the Illumina Hiseq X Ten system, from six early developmental stages (DS) (Additional file 1: Figure S1) of *H. nobilis*, with five replicates of each stage. A total of 1,498,510,466 clean reads (224 Gb) were obtained from the 30 cDNA libraries following quality control. The percentage of valid bases, the G + C content, and fraction of bases with quality scores of Q30 for the 30 libraries were an average of 95.00, 46.45, and 92.40%, respectively (Additional file 2: Table S1). After de novo assembly and the removal of redundancy, in which sequences < 300 base were not taken into account, the clean reads were used for further downstream analyses. A total of 76,573 unigenes were assembled from the cleaned sequence reads, with an average length of 1768 base (minimum 301 base and maximum 32,074 base), and an N50 length of 2834 base (Additional file 3: Table S2). The length distributions of all the assembled unigenes are shown in Additional file 4: Figure S2a, with 30.84% (23,616) of all unigenes being longer than 2 kb.

The FPKM (Fragments Per Kilobase of transcript per Million mapped reads) method was used to estimate gene expression. The distributions of FPKM values for each sample are shown using a box-plot and stack-plot in Additional file 4: Figure S2b and S2c. The results showed that the transcription of embryonic genes became increasingly more active from DS1 to DS2. More unigenes showed a low-expression level in DS1 (FPKM < 1) compared with that in later development stages. However, similar amounts of unigenes (5889 – 8122) with a high-expression level (FPKM ≥10) were detected in all libraries.

### Annotation, functional classification, and coding sequence (CDS) prediction

**Fig. 1:**
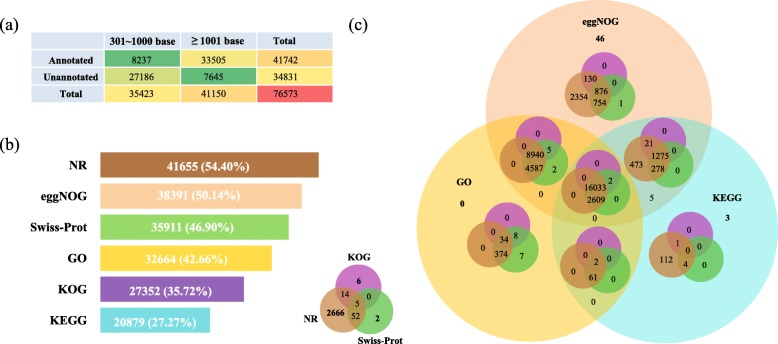
Tabulated the annotation distribution counts in different length ranges of unigenes (**a**). Bar chart (**b**) and Venn diagram (**c**) of the annotation statistics for unigenes mapped to public databases

The assembled sequences were used as queries in BLAST searches (≤ e^− 5^) against the non-redundant (NR), Swiss-Prot, gene ontology (GO), Kyoto Encyclopedia of Genes and Genomes (KEGG), Eukaryotic orthologous groups (KOG), and evolutionary genealogy of genes: Non-supervised Orthologous Groups (eggNOG) databases. Ultimately, more of the longer unigenes were annotated (Fig. 1a). The statistic of the annotation results for the databases are shown in Fig. 1b and Fig. 1c. The majority of the unigenes (41,655; 54.40%) were mapped to the NR database. In contrast, only 20,879 (27.27%) annotated unigenes were matched to the KEGG database. In brief, 41,742 (54.51%) unigenes were annotated, with 16,033 (20.94%) unigenes being synchronously annotated in all six databases.

The outcome of the homology search of unigenes in the NR databases showed that all of the top ten matching species were teleosts (Additional file 5: Figure S3a). Unigenes were assigned to the three principal GO database classification domains, i.e., “cellular component”, “molecular function”, and “biological process” (Additional file 5: Figure S3b). Unigenes showing homology to annotated members of KEGG pathways were divided into five ontology categories, and the subcategories of “signal transduction” and “endocrine system” contained the most genes (Additional file 5: Figure S3c). The annotated unigenes were initially classified into 25 KOG categories, with the two largest groups being “signal transduction mechanisms” and “general function prediction only” (Additional file 5: Figure S3d). The majority of the unigenes were matched to the class of “function unknown” in the eggNOG database (Additional file 5: Figure S3e). We detected 39,680 coding sequences (CDSs) using BLAST against the database, and 6084 novel potential CDSs were predicted (Additional file 5: Figure S3f).

### Correlation and principal component analysis of the samples

**Fig. 2:**
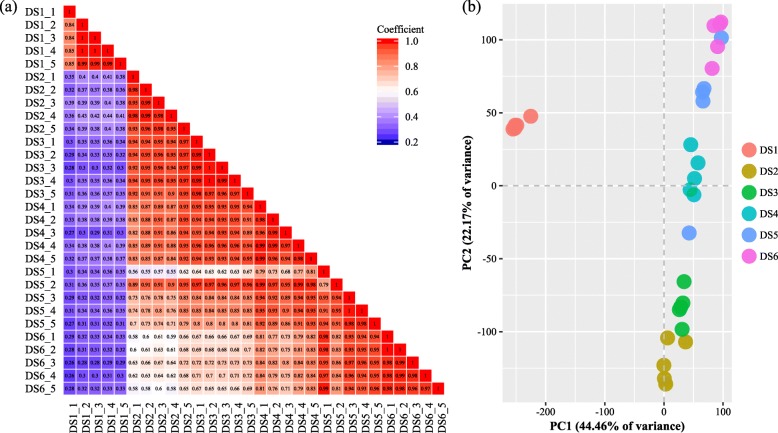
The correlation coefficients matrix and heatmap among samples (**a**) and the principal component analysis plot (**b**), based on the FPKM data (mean ≥ 1)

To evaluate the consistency of the sample collection and investigate the transcriptomic relationship among the sampling stages, we performed correlation and principal component analysis (PCA) for the samples using the transcript expression data (mean FPKM ≥1). Both analyses could provide an overview of the reliability of the experimental results and the rationality of sampling. The Pearson correlation coefficient between the five biological replicates of the six groups in this study had very high repeatability, i.e., all *R* > 0.79 (Fig. 2a). The PCA plot (Fig. 2b) was in agreement with the correlation analysis results. Most samples from the same stages were grouped together excepted for two samples of DS5 (DS5_1 and DS5_2), which were removed from the subsequent comparison analyses.

### Pairwise differentially expressed transcripts (DETs), enrichment, and protein-protein interaction (PPI) analysis

**Fig. 3:**
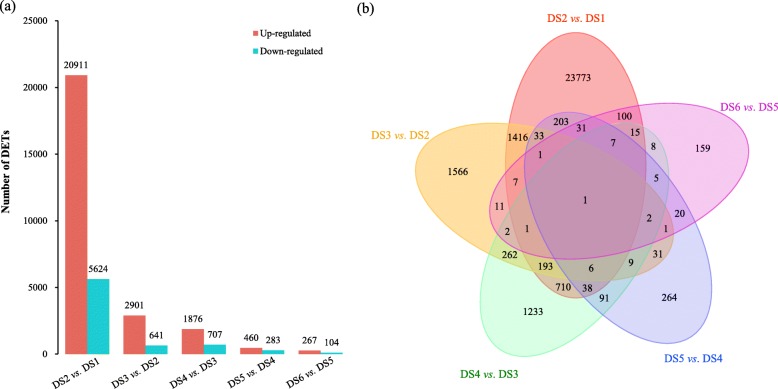
Bar chart (**a**) and Venn diagram (**b**) statistics for differentially expressed transcripts (DETs) identified from pairwise comparison of adjacent development stages in *H. nobilis*

In this study, a total of 30,199 DETs were confirmed (fold change > 4 or < 0.25, and false discovery rate PDR < 0.01) in comparisons between adjacent developmental stages of *H. nobilis*. The statistical information and Venn diagram of the pairwise comparative DETs are shown in Fig. 3. Among the comparisons, DS2 vs. DS1 showed the biggest difference, with 20,911 up-regulated and 5624 down-regulated DETs. At the global level, detection according to a strict dynamic decrease of the difference in developmental ordering, indicated that the changes between earlier adjacent stages were much larger than those between later adjacent stages. This result was consistent with the sample correlation and PCA analyses using the transcript subset (mean FPKM ≥1). Generally, the number of up-regulated transcripts was higher than the number of down-regulated transcripts for each comparison, especially in the earlier stages.

**Fig. 4:**
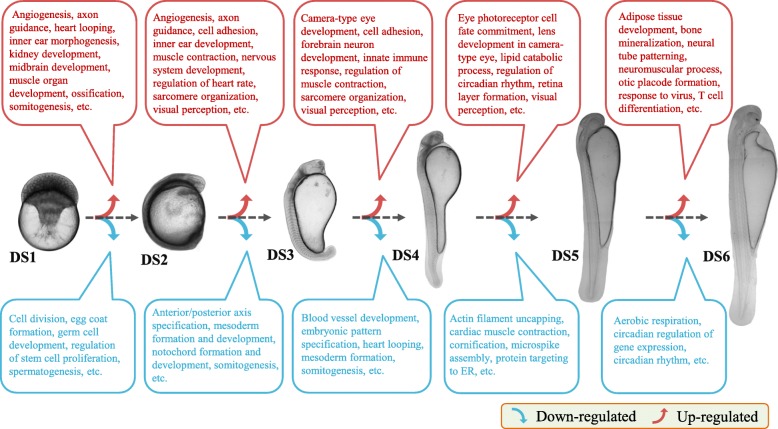
Differentially regulated GO (gene ontology; biology process) terms enriched for differentially expressed transcripts (DETs) identified from pairwise comparison of adjacent development stages in *H. nobilis*

**Fig. 5:**
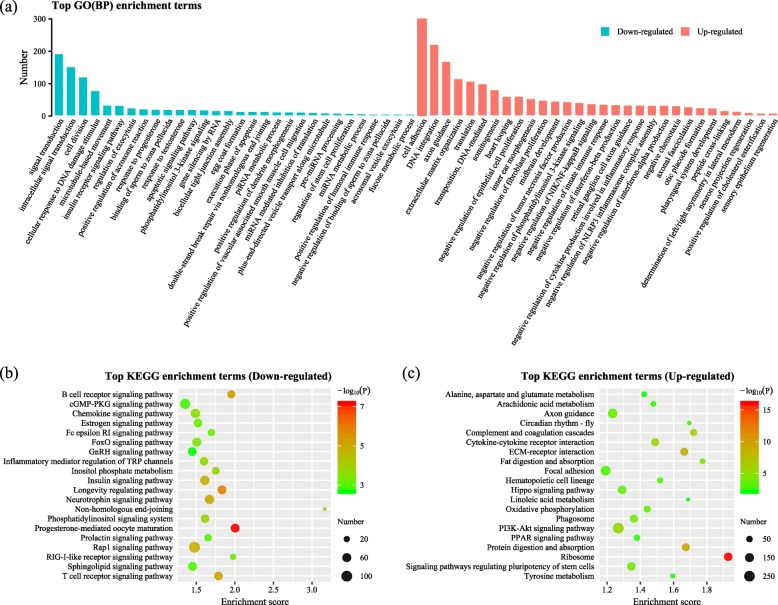
The top GO (gene ontology; biology process) (**a**) and Kyoto Encyclopedia of Genes and Genomes (KEGG) pathway (**b**, and **c** for down- and up-regulated, respectively) enrichments for the differentially expressed transcripts (DETs) detected in the DS2 vs. DS1 comparison

The top GO function (biological process, BP) and KEGG pathway enrichment terms for the pairwise comparisons (up- and down-regulated) between adjacent development stages are shown in Additional file 6: Table S3. Based on the GO (BP) terms enriched, we constructed a rough transcriptomic dynamic map for the early developmental period in the *H. nobilis* (Fig. 4). There are some biological processes (related to ontogenesis, organogenesis, morphological change, immune and nervous system development, etc.) that were differentially regulated in certain periods. In view of the largest difference detected between DS2 and DS1, the top specific GO (BP) and KEGG pathway enrichments in DS2 vs. DS1 were plotted in Fig. 5. We noticed that several biological processes (egg coat formation, gene silencing by RNA, miRNA mediated inhibition of translation, and piRNA metabolic process, etc.) and metabolic pathways (estrogen signaling pathway, progesterone-mediated oocyte maturation, and prolactin signaling pathway) might be related to maternal mRNA expression and degradation in the down-regulated DETs in the pairwise comparison. In contrast, the abundance up-regulated DETs enriched in “DNA integration”, “translation” biological processes, and “ribosome” pathway, might predict the activation and translation of zygotic mRNA in this developmental period.

**Fig. 6:**
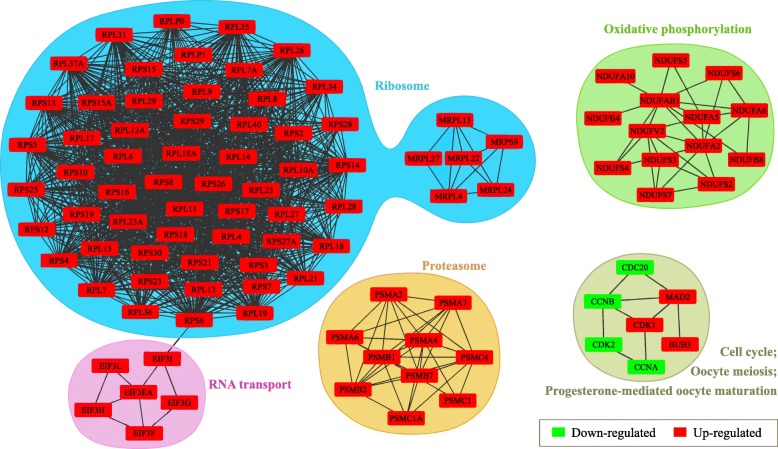
The protein-protein interaction (PPI) networks structured for the differentially expressed transcripts (DETs) identified from the DS2 vs. DS1 comparison, based on the top combination cores with the STRING database

Based on the pairwise DETs in DS2 vs. DS1, the (combination core) known PPIs from the STRING database were used to construct PPI networks (Fig. 6). The genes (or proteins) from the same pathway or related pathways were integrated together, and some of those pathways were detected among the KEGG enrichment terms (i.e., “ribosome”, and “oxidative phosphorylation” pathways were enriched in the up-regulated terms; and “progesterone-mediated oocyte maturation” pathways was enriched in the down-regulated terms, as shown in Fig. 5).

### Series-cluster analysis, enrichment, and co-expression analysis

**Fig. 7:**
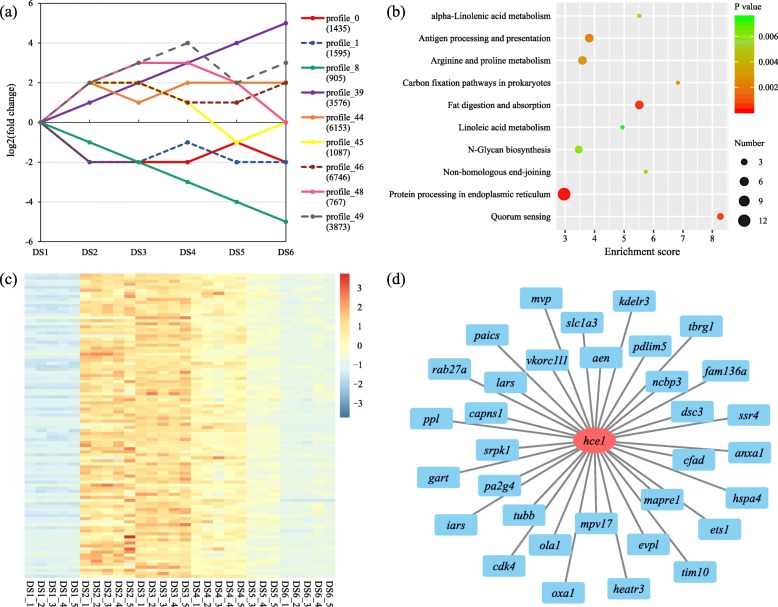
Nine series-cluster profiles (**a**) simulated for the differentially expressed transcripts (DETs) expression tendency. The Kyoto Encyclopedia of Genes and Genomes (KEGG) enrichment for unigenes of profile_48 (**b**), a heatmap constructed for top 100 unigenes (calculated based on mean Fragments Per Kilobase of transcript per Million mapped reads, FPKM of DS3 and DS4 stages) from profile_48 across developmental stages (**c**), and the co-expression network shows the genes co-regulated with hatching enzyme gene (*hce1*) (**d**)

To analyze the transcripts’ expression trends during the early development period, a total of 30,199 DETs were classified into nine simulation profiles (*P* < 0.05) based on the mean FPKM values of the unigenes in each stage (Fig. 7a). The specific profiles such as continuously decreasing (profile_8), continuously increasing expression (profile_39), and oscillatory expression (profile_48) were identified, which implied that during the early developmental period, gene expression is strictly regulated. We noticed that profile_48 might be related to hatching in the early development of *H. nobilis*, which showed a gradient increase before (DS1, DS2, and DS3) and during (DS4) the hatching stages, and then decreasing during the post-hatching stages (DS5, and DS6).

The top GO (BP) and KEGG pathway enrichment terms for different series-cluster profiles are shown in Additional file 7: Table S4. For profile_48, the largest number of unigenes were enriched with “protein processing in endoplasmic reticulum” (Fig. 7b), which is an important pathway for the transport of proteins, which might relate to the secretion of hatching related enzyme.

Based on the mean FPKM values of ten samples from around hatching (DS3 and DS4 stages) in profile_48, the top 100 unigenes with higher expression were chosen for further analysis, and the heatmap for the expression of these unigenes showed highly similar trends (Fig. 7c). Among them, three unigenes (CL28971Contig1, comp42501_c0_seq1_6, and comp65497_c0_seq1_1) were annotated as a hatching enzyme gene (*High choriolytic enzyme 1*, *hce1*) by matching to the NR database. There was no available PPI information related to HCE in the STRING database. The co-expression network of 33 other annotated genes related to *hce1* was constructed based on Pearson correlation analysis (*R* > 0.85, *P* < 0.01) among expression levels in different samples of all stages (Fig. 7d). These genes were predicted to be related to the hatching during embryo development in *H. nobilis*.

### Validation of RNA-seq data using quantitative real-time PCR (qPCR)

To verify the RNA-seq expression data, 12 unigenes displaying diverse expressions between adjacent stages were selected for qPCR validation. Among them, 10 unigenes were detected with significantly up regulated expression trends (fold change > 2, *P* < 0.05) both by the T-test (one-tail) and the Wilcoxon test (one-tail) (Additional file 8: Table S5). The significant positive correlations (*R* = 0.68 for all unigenes, *P* < 0.01; and *R* = 0.76 for 10 unigenes, *P* < 0.01) were observed between the RNA-seq and qPCR data (log-transformed) (Additional file 9: Figure S4), which confirmed the high reliability of the RNA-seq data obtained in the present study.

### Simple sequence repeat (SSR) analysis

In addition, 59,014 SSR (or microsatellite) loci were identified among the unigenes, the most abundant SSR loci were mono-nucleotide repeats type (34,580, 58.60%), followed by dinucleotide repeats (10,882, 18.44%), and trinucleotide repeats (4478, 7.59%) (Additional file 10: Figure S5).

## Discussion

In terms of the long unigene count (> 2000 base), the average length, and the annotation percentage, the de novo assembly presented in this study was of higher quality than those presented in previous transcriptomic studies in *H. nobilis* [11, 12]. This suggested that our data could provide effective genetic information, especially for those genes or enriched terms concerned with the early development of this fish. The unigene count detected in this study fell in the range of counts reported in two previous studies [11, 12]. Compared with the transcriptomic studies using microarrays or a genome assembly [14–16], large number of unigenes were detected in transcriptomic studies in *H. nobilis* [12] and other fish species [18, 22]. In our study, the reason for this large number of transcripts might be that the transcriptomes were assembled from mixed libraries of multiple samples (as no genome reference of this species is available), the isoforms and single nucleotide variation would lead to more unigenes being predicted. The unigene count difference detected among these studies, might be due to the differences in sampling method, sequencing depth, and sequencing platform used. Homology searching using the NR databases showed that all of the top ten matching species were teleosts (Additional file 5: Figure S3a). The qPCR results validated the high reliability of the transcriptome data and the DETs analyses (Additional file 9: Figure S4). Abundant SSR loci were detected among the unigenes (Additional file 10: Figure S5), which would provide more potential molecular markers for genetic studies in *H. nobilis*.

In the present study, the gene expression pattern identification and comparative transcriptome analyses were carried out for early development stages in *H. nobilis*. The correlation and PCA results for the samples demonstrated high homogeneity among the samples from the same stage, especially in the early embryo stages (Fig. 2). Two samples (DS5_1, and DS5_2) overlapped with the adjacent stages, which might be related to the group sampling from mass spawning, as different spawning times are commonly present in brooders. Furthermore, it is difficult to choose the samples with few phenotypic changes after hatching, which was why we used the time series for sampling after hatching. Therefore, those two samples were removed from further comparative analyses. At the global level, the expression level of the unigenes increased obviously after the DS1 stage (early blastula) (Additional file 4: Figure S2), and the numbers of DETs gradually declined following the continuous development of embryos and larvae (Fig. 3), which indicated that the largest transcriptomic dynamic change occurred during the period from DS1 to DS2 stages. For each pairwise comparison, the enriched biological process and pathway terms of down- and up-regulated conditions indicated the change trends in the given periods, and some biological processes related to ontogenesis, organogenesis, morphological change, and immune and nervous system development were regulated differently in different development stages (Fig. 4). Those DETs of enrichment terms with high expression levels may also represent special or important biological events during the early and latter stages. The corresponding genes are potential differentiation biomarkers that could be useful in developmental studies and could be analyzed using in situ hybridization in embryos or larvae. Meanwhile, we identified nine stimulation profiles for expression patterns, one of which (profile_48) might be strictly related to the hatching event (Fig. 7a). The results confirmed that embryogenesis is a highly dynamic process in terms of gene expression and relative regulation in *H. nobilis*.

The largest difference in gene expression was detected between DS1 and DS2, which might be related to the maternal to zygotic transition (MZT). As reported in the studies of zebrafish embryogenesis, MZT mostly occurs from the blastula to gastrula stages [13, 16]. The sampled period between DS1 and DS2 in this study, was speculated to encompass the MZT in *H. nobilis*. Therefore, the specific and highly expressed unigenes detected in the DS1 stage, could be regarded as maternal transcripts in *H. nobilis*. The biological processes in the early stages of vertebrate development rely exclusively on maternal transcripts (maternal mRNAs) [23, 24], and maternal mRNAs are also responsible for the zygote genome activation (ZGA) during the MZT [25]. A proportion of the maternal mRNAs are essential for normal embryogenesis and development in fish. Many studies have reported abnormal embryo development when maternal mRNAs are inhibited or disturbed, such as in rainbow trout (*Oncorhynchus mykiss*) [26] and zebrafish [27]. Besides, maternal RNAs were identified that correlated with the hatching success and embryo quality of Atlantic halibut (*Hippoglossus hippoglossus*) [28, 29], and the embryonic mortality of Atlantic cod (*Gadus morhua*) [30]. Therefore, those genes enriched in terms of maternal biological processes and pathways (Fig. 5) could be used in further research for their biological function during early development and the relationship with egg quality or embryonic mortality in *H. nobilis*.

Genes with abundant expression that were down-regulated significantly between DS1 and DS2 might represent maternal transcripts that were degraded early in development. Transcript clearance during the MZT is necessary for development [31], and the deadenylation process plays key role in triggering mRNA degradation during embryonic development [32]. Although the mechanism behind deadenylation is unclear, active deadenylation and subsequent decay of maternal transcripts was detected in zebrafish [13]. In *Drosophila* [33] and zebrafish [34], this process relies on Piwi-associated RNAs (piRNAs) and their associated proteins. For the down-regulated DETs in the DS2 vs. DS1, the GO and KEGG analyses identified processes that might be related to the maternal mRNA degradation during MZT of *H. nobilis*, e.g., the biological processes of “gene silencing by RNA”, “miRNA mediated inhibition of translation”, “piRNA metabolic process”, and “RNA degradation” pathway. The onset of the ZGA is tightly coordinated with maternal transcripts degradation in zebrafish [16]. In contrast, else abundant transcripts that were up-regulated from DS1 to DS2 might be related to ZGA, which is predicted to take place in this developmental period in *H. nobilis*. That represents a reasonable interpretation for the increase in global expression from DS1 to the later stages (Additional file 4: Figure S2b and Figure S2c). According to the functional annotation, the “translation” biology process and “ribosome” pathway are enriched for up-regulated DETs in the period from DS1 to DS2, and are probably related with ZGA of early development in *H. nobilis*. A similar overrepresentation of the ribosomal pathway was found previously in zebrafish [16]. The high expression of ribosomal transcripts most likely occurs because ribosomal proteins are necessary to assemble the ribosome, which serves as the site of protein synthesis.

Co-expressed genes (proteins) are usually members of the same pathway or protein complex, which are functionally related or controlled by the same transcriptional regulatory program [35]. We noticed that the proteins from the same or related metabolic pathways were distinctly integrated together in PPI networks during the period from DS1 to DS2 (Fig. 6). We speculated that those proteins (or genes) could be co-regulated during the early development of *H. nobilis*. Following fertilization, maternal genes such as cyclin dependent kinase (CDK), mitotic arrest deficient 2 (MAD2), and BUB3 mitotic checkpoint protein (BUB3) are necessary for basal cellular processes in mammals, including the completion of meiosis, resumption of meiotic progression and proper chromatin and spindle regulation [36]. Those genes mapped in the PPI networks in this study, that were enriched with “cell cycle”, “oocyte meiosis”, and “progesterone-mediated oocyte maturation” pathways might be related to reproductive potential in *H. nobilis*. The PPIs among ribosomal proteins (RPs) detected in the present study (enriched in the “ribosome” pathway) might be related to the ribosome assembly process, which involves electrostatic and hydrogen bonding interactions [37]. In addition to their important roles during the ribosome biogenesis, many RPs are believed to be essential for the normal embryonic development in zebrafish [38, 39]. The eukaryotic translation initiation factor 3 complex (eIF3, enriched in the “RNA transport” pathway) plays important roles in protein synthesis, and is essential for embryonic development and cell proliferation [40]. The mitochondrial ribosome proteins (MRPs, enriched in the “ribosome” pathway) are essential for mitochondrial ribosome assembly, and offer translation sites for mitochondrial DNA genes. Furthermore, the integrated process of oxidative phosphorylation for ATP production (enriched in the “oxidative phosphorylation” pathway) is executed in the mitochondria, which meets most of the energy requirements for cell growth, differentiation, and development. In humans, the down-regulation of mitochondrial RNA expression caused by defective oxidative phosphorylation genes possibly affects oocyte quality, the fertilization rate, and further embryo development [41], and loss or mutation of MRPs are reported to result in mitochondrial diseases [42]. However, whether the MRPs have similar effects in fish is unknown. The remaining proteins were enriched in “proteasome” pathway in the PPI networks, which also reported play important roles to modulate ZGA and maternal mRNA degradation in embryogenesis and development [43].

In this study, we identified a remarkable gene expression profile that might be related to the hatching during *H. nobilis* early development (profile_48, Fig. 7a). Hatching is an important process for the early development in oviparous fishes, in which hatching enzymes (HEs) play crucial roles [44]. Two kinds of HE homolog are reported to cooperatively digest the egg envelope in medaka (*Oryzia latipes*) [45], including the high choriolytic enzymes (HCEs) and low choriolytic enzymes (LCEs). The expression patterns of HEs and HE genes during embryonic development have been studied in several fish species [17, 44–46]; however, the mechanisms and pathways involved remain poorly understood. In the present study, the largest enriched pathway was “protein processing in endoplasmic reticulum (ER)” (Fig. 7b). The ER serves many general functions, including the folding and transport of synthesized proteins. The expression patterns of unigenes coincided closely with that of *hce1* in profile_48, which showed high expression in the hatching stages (Fig. 7c). The results indicated many genes are co-regulated with *hce1*, and might be related to HE processing and secretion during embryogenesis. Among those genes detected in the co-expression analysis (Fig. 7d), *ssr4* encodes a subunit of TRAP (translocon-associated protein) complex that is necessary for the efficient translocation of nascent proteins across or integrated into the ER lumen [47]. Another subunit of the TRAP complex (TRAPβ) in zebrafish embryogenesis was reported to show enhanced expression in hatching gland precursors [48]. The *hspa4* (*hsp70*) gene encode molecular chaperones (Hsp70) that play crucial roles in protein folding [49]. Only properly folded proteins are transported from the ER to the Golgi apparatus. The *kdelr3* gene encodes a receptor for the C-terminal sequence motif KDEL (Lys-Asp-Glu-Leu) that is present on ER resident proteins and that mediates their recycling from the Golgi back to the ER [50]. However, to the best of our knowledge, they have not associated with a pathway (or network) related with hatching in fish species. The genes co-expressed with *hce1* detected in this study could potential genetic resources (or biomarkers) for further research. Moreover, a unigene (CL37Contig19) annotated as encoding an LCE, was not clustered into profile_48, and showed low expression in the analyzed samples. This implied that hatching of *H. nobilis* embryos is probably controlled by a single enzyme (HCE), similar to zebrafish [46]. In addition, the LCEs were reported to have lower and more stable expression levels compared with HCEs [17], and may affect the hatching process in embryogenesis later than HCEs [45].

## Conclusions

The present report is the first to describe combined transcriptome profiling of the early developmental stages in *H. nobilis*. The results indicated a strict dynamic regulation of early development in this species, especially before hatching. This study provides valuable new transcriptomic information and resources related to early development in *H. nobilis*. The largest difference in gene expression was detected might be related to MZT in embryogenesis, and showed distinctly PPI in enrichment metabolism pathways. We identified one remarkable gene expression profile (profile_48) might be related to the hatching during *H. nobilis* early development, and constructed a co-expressed network consisted *hce1* and 33 other annotated genes from this profile. Meanwhile, abundant novel SSR loci were detected, which could represent molecular markers for genetic research in this species. However, this study on the early life history of *H. nobilis* used quite distant sample points, as well as pools of embryos or larvae for each stage; further research is still required, such as the use of a high-resolution time series and single individual sampling in embryogenesis.

## Methods

### Animal experiment and sample collection

Culturing and spawning of *H. nobilis* brooders were carried out at the Suzhou Wei-Lai Aquatic Breeding Farm (Provincial hatchery of Jiangsu, China). Twelve pairs of wild adult fish from Yangtze River were used for artificial breeding during May 15, 2016. Brood fish were processed under hormone stimulation (chorionic gonadotrophin for injection, dosage with 1200 IU/kg for female, and 800 IU/kg for male) and mass spawning in a round spawning pond (diameter with 8 m, and depth with 1.5 m). The brood fish were cultured in pond for recovery after the study, and released finally into wild. Fertilized eggs were collected and hatched in a cylinder, with continuous fresh water at 22–24 °C.

For transcriptome sequencing, total of 375 specimens of 30 samples (15 specimens/sample for three embryonic stages, ten specimens/sample for three larval stages, with five biological replicates at each stage) were periodically collected at six early developmental stages of *H. nobilis*. The rest of embryos and larvae were put back for further hatching and nursing, and released finally into wild for resource enhancement. The stages included early blastula (DS1, about 3.5–5 h post-fertilization (hpf)), 6-somite (DS2, about 15–16 hpf), 26-somite (DS3, about 23–24 hpf), hatching (DS4, about 29–30 hpf), 12 h post-hatching (hph) (DS5), and 24 hph (DS6) stages. The embryos and larvae were monitored under a microscope to ensure developmental synchrony. Samples at the same developmental stage were collected, and immediately washed with phosphate-buffered saline (PBS) and stored in RNAstore Reagent (Tiangen, Beijing, CA, China), followed by freezing at − 80 °C until RNA extraction.

### RNA extraction, cDNA library construction, and sequencing

Total RNA was extracted from whole organisms of the above samples using a mirVana miRNA Isolation Kit (Ambion, Austin, TX, USA) following the manufacturer’s protocol. The total RNA content of each sample was measured using a NanoDrop 2000c UV Spectrophotometer (Thermo Fisher Scientific Inc., Waltham, MA, USA), and the quality of the RNA was assessed using agarose gel electrophoresis and an Agilent Bioanalyzer 2100 system (Agilent Technologies, Santa Clara, CA, USA). The samples with an RNA Integrity Number (RIN) > 7were retained for subsequent analysis.

The libraries were constructed using a TruSeq Stranded mRNA LTSample Prep Kit (Illumina, San Diego, CA, USA) according to the manufacturer’s instructions. Briefly, 4 μg of total RNA was used as the starting material for library preparation with the Illumina TruSeq RNA preparation Kit. Poly-T oligo-attached magnetic beads were used to purify the mRNA, which was then fragmented at 94 °C for 8 min. First-strand and second-strand cDNA were subsequently synthesized. The double stranded cDNA was then purified for end repair, dA tailing, adaptor ligation, and DNA fragment enrichment. The AMPure XP system (Beckman Coulter, Beverly, CA, USA) was used to purify the library fragments. The library quality was assessed using an Agilent 2100 Bioanalyzer system. The libraries were then sequenced by Shanghai OE Biotech (Shanghai, China) on an Illumina Hiseq X Ten sequencing platform to generate the 150 bp paired-end reads. All sequencing reads were exported in the FASTQ format. The sequence data were deposited in the NCBI Sequence Read Archive (SRA) under the accession numbers SRP143548.

### Transcriptome assembly, functional annotation, and CDS analysis

All 30 libraries were used to assemble a comprehensive transcriptome reference representing the six developmental stages. Raw data (raw reads) were processed using Trimmomatic [51]. The reads containing Poly-N and the low quality reads were removed to obtain the clean reads. After removing adaptors and low quality sequences, the clean reads were de novo assembled into transcripts using Trinity (version: trinityrnaseq_r20131110) [52] with the paired-end method. For each assembly, the longest transcript was chosen as a unigene based the similarity and length of the sequence for subsequent analysis.

The function of the unigenes was annotated by their alignment with the NR, GO, Swiss-Prot, KOG, eggNOG, and KEGG databases using BLASTX [53] with a threshold E-value of 10^− 5^. The proteins with the highest hits to the unigenes were used to assign the functional annotations. Based on the Swiss-Prot annotation, GO classification was performed by the mapping relation between Swiss-Prot and GO terms. The unigenes were mapped to the KEGG database to annotate their potential metabolic pathways. When a unigene to be appeared unaligned to any of the databases, the ESTScan software [54] was applied to predict its CDS.

### Identification of DETs and series-cluster analysis

High quality sequencing reads were mapped to the assembled reference using Bowtie 2 [55]. The output was analyzed using the RSEM software [56] to obtain expected counts (Additional file 11: Table S6) and FPKM values. The statistics for FPKM value of unigenes, hierarchical clustering (Pearson correlation), and PCA (Principal component analysis) for the 30 samples used in the present study were performed using stats, reshape2, dplyr, and ggplot2 packages in the R program (http://www.R-project.org/).

The EBSeq package [57] was applied to call for DETs, using the classic method with five iterations. Parameters were set as fold change > 4 (up-regulated) or < 0.25 (down-regulated), and the adjusted *P* value (FDR) < 0.01 as the threshold to define significant differences. The Venn diagram was depicted using the VennDiagram package in the R program.

Series-cluster analysis was performed using STEM [58] to classify the differentially expressed genes in different clusters based on the FPKM change tendency of the genes in the six development stages, set with default parameters. A heatmap of genes expression was constructed using the pheatmap package in the R program.

### Enrichment analysis, PPI, and co-expression analysis of DETs

The enrichment analysis of GO and KEGG pathways was performed based on the DETs identified from pairwise comparisons and series-cluster profiles, which were performed using the R program based on the hypergeometric distribution. Fisher’s exact test was applied to identify the significant terms (GO or KEGG categories). The threshold of significance was defined by *P*-value < 0.01. The enrichment score was calculated by using: Enrichment score = (n_f_/n)/(N_f_/N), where “N” was the number of the background genes, “n” was the number of total DETs or the genes analyzed with annotation, “n_f_” and “N_f_” represented the DETs and background genes with the target annotation terms, respectively. The DETs were imported into the STRING database (https://string-db.org/) to obtain the know PPI networks [59], which was constructed using the STRING app in the Cytoscape software [60].

Gene co-expression network analysis was performed to track the interactions among the DETs in specific profile, according to their dynamic expression changes in the six developmental stages. A Pearson correlation matrix among genes was calculated using the stats package in the R programs, and the significantly correlated pairs (*R* > 0.85, *P* < 0.01) were selected to construct the co-expression network using Cytoscape software [60].

### Quantitative real-time PCR (qPCR)

Six up-regulated DETs from DS2 vs. DS1 (CL117Contig2, CL154Contig3, CL18044Contig1, CL4103Contig1, CL7347Contig1, and comp35037_c0_seq1_6), six up-regulated DETs from DS4 vs. DS3 (CL10120Contig2, CL11117Contig1, CL18291Contig1, CL5332Contig1, CL8548Contig1, and comp15897_c0_seq1_5) and *actb* gene (encoding β-actin, comp37248_c0_seq1_6) were selected and used to validate the transcriptome data via qPCR. The primer pairs (Additional file 8: Table S5) were designed based on the unigene sequences, and then synthesized by Generay Biotech Co., Ltd. (Shanghai, PRC).

Real-time PCR was performed using a LightCycler® 480 II Real-time PCR Instrument (Roche, Basel, Switzerland) with 10 μl of PCR reaction mixture that included 1 μl of cDNA, 5 μl of 2× QuantiFast® SYBR® Green PCR Master Mix (Qiagen, Germany), 0.2 μl of each primer (10 μM) and 3.6 μl of nuclease-free water. Reactions were incubated in a 384-well optical plate (Roche) at 95 °C for 5 min; followed by 40 cycles of 95 °C for 10 s, 60 °C for 30 s and 72 °C for 30s. Each sample was run in triplicate for analysis. At the end of the PCR cycles, melting curve analysis was performed to validate the specific generation of the expected PCR product. Relative expression was calculated using the 2^-(ΔΔCt)^ method with *actb* gene as the reference control [61].

The Shapiro-Wilk normality test and Levene’s test for homogeneity of variances was carried out using the stats and car packages in the R program, respectively. The T-test (one-tail), and Wilcoxon test (one-tail) for difference comparison (qPCR) between stages (DS2 vs. DS1, or DS4 vs. DS3) were performed using the stats package in the R program. In correlation analysis, concerned to the large variances between stages, the relative expression (qPCR) and FPKM values were corrected using logarithmic transformation. To avoid arithmetic error and large negative values in transformation, a pseudo-count of 1 was added to each value before log-transformation. Similar methods were reported in other studies [62–65]. The scatter plots were visualized using Microsoft excel. The Pearson correlations were calculated using the stats package in the R program.

### Simple sequence repeat (SSR) marker discovery

SSRs in the transcriptome were identified using MISA (http://pgrc.ipk-gatersleben.de/misa/). The motif unit/minimum repeats were set as: 1 base/10 repeats, 2 base/6 repeats, and 2–6 base/5 repeats. The interruptions (max interval between two SSRs) set to 100 bases for compound loci identification.

## Supplementary information

**Additional file 1: Figure S1.** Six developmental stages sampled in *H. nobilis*.

**Additional file 2: Table S1.** The specific statistics for each sequencing library and quality control.

**Additional file 3: Table S2.** The summary information of de novo assembling.

**Additional file 4: Figure S2.** The length distribution of unigenes assembled in the early developmental stages of *H. nobilis* (a). The boxplot of Fragments Per Kilobase of transcript per Million mapped reads (FPKM) values (b), and the expression distribution counts in different levels of unigenes in each library (c).

**Additional file 5: Figure S3.** The annotation statistics of unigenes from five public databases (non-redundant (NR), gene ontology (GO), Kyoto Encyclopedia of Genes and Genomes (KEGG), Eukaryotic orthologous groups (KOG), and evolutionary genealogy of genes: Non-supervised Orthologous Groups (eggNOG), respectively) (a-e), and the coding sequence (CDS) detection and prediction result (f).

**Additional file 6: Table S3.** The top GO (gene ontology; biology process) and Kyoto Encyclopedia of Genes and Genomes (KEGG) enrichment terms for the differentially expressed transcripts (DETs) identified from pairwise comparisons of adjacent developmental stages in *H. nobilis*.

**Additional file 7: Table S4.** The top GO (gene ontology; biology process) and Kyoto Encyclopedia of Genes and Genomes (KEGG) enrichment terms for unigenes from different expression profiles during the early development in *H. nobilis.*

**Additional file 8: Table S5.** The primers, quantitative real-time PCR (qPCR) data, and Fragments Per Kilobase of transcript per Million mapped reads (FPKM) values for twelve unigenes chosen for verification.

**Additional file 9: Figure S4.** The correlations of quantitative real-time PCR (qPCR) and Fragments Per Kilobase of transcript per Million mapped reads (FPKM) data of all twelve chosen unigenes (a) and the ten unigenes with coincident expression trend (b).

**Additional file 10: Figure S5.** The statistic of simple sequence repeat (SSR) loci detected in the unigenes from transcriptome sequencing.

**Additional file 11: Table S6.** The expected counts for unigenes detected across developmental stages in *H. nobilis*.

## Data Availability

The dataset supporting the conclusions of this article is available in the NCBI and Sequence Read Archive (SRA) repository, accession number SRP143548 (Release data: 2019-08-15) https://www.ncbi.nlm.nih.gov/sra/SRP143548.

## Abbreviations

BP: Biological process
BUB3: Mitotic checkpoint protein BUB3
CAFS: Chinese Academy of Fishery Sciences
CDK: Cyclin-dependent kinase
DET: Differential expressed transcript
eIF3: Eukaryotic translation initiation factor 3 complex
ER: Endoplasmic reticulum
FFRC: Freshwater Fisheries Research Center
FPKM: Fragments per kilobase of transcript per million fragments mapped
GO: Gene ontology
HCE: High choriolytic enzyme
HE: Hatching enzyme
*hsp*: heat shock protein gene
*kdelr3*: KDEL (Lys-Asp-Glu-Leu) endoplasmic reticulum protein retention receptor 3
KEGG: Kyoto Encyclopedia of Genes and Genomes
KOG: Eukaryotic Orthologous groups
LCE: Low choriolytic enzyme
MAD2: Mitotic arrest deficient 2
MRP: mitochondrial ribosome protein
MZT: Maternal-zygotic transition
NCBI: National Center for Biotechnology Information
NR: Non-redundant
PCA: Principal component analysis
PPI: Protein-protein interaction
q-PCR: Quantitative real-time PCR
QTL: quantitative trait locus
RIN: RNA integrity number
RNA-Seq: RNA-Sequencing
RP: Ribosomal protein
SSR: Simple sequence repeat
*ssr4*: Translocon-associated protein subunit delta
TRAP: Translocon-associated protein
ZGA: Zygotic genome activation
