# Supplementary figures and images for: Dynamic transcriptome sequencing and analysis during early development in the bighead carp (Hypophthalmichthys nobilis)
Source: BMC Genomics. 2019 Oct 28;20:781. doi: 10.1186/s12864-019-6181-4 (PMC6819325; doi:10.1186/s12864-019-6181-4)

**DS1**

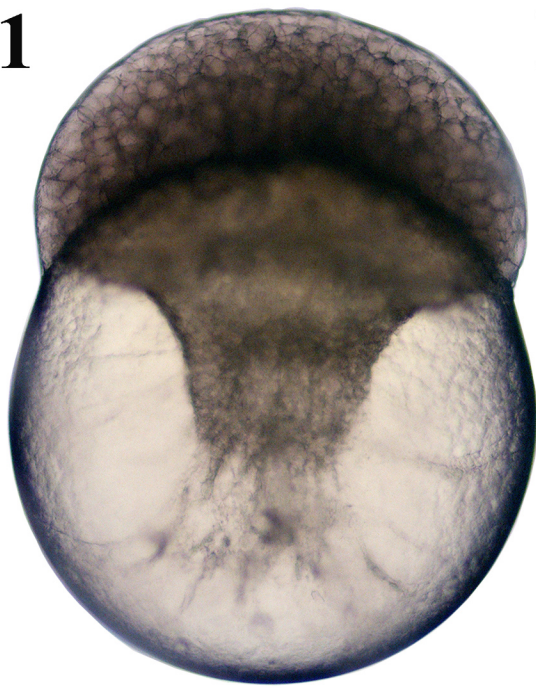

**DS2**

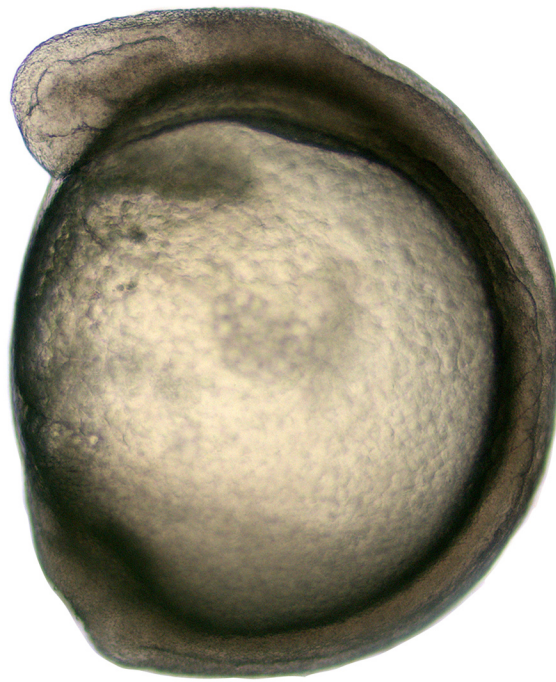

**DS3**

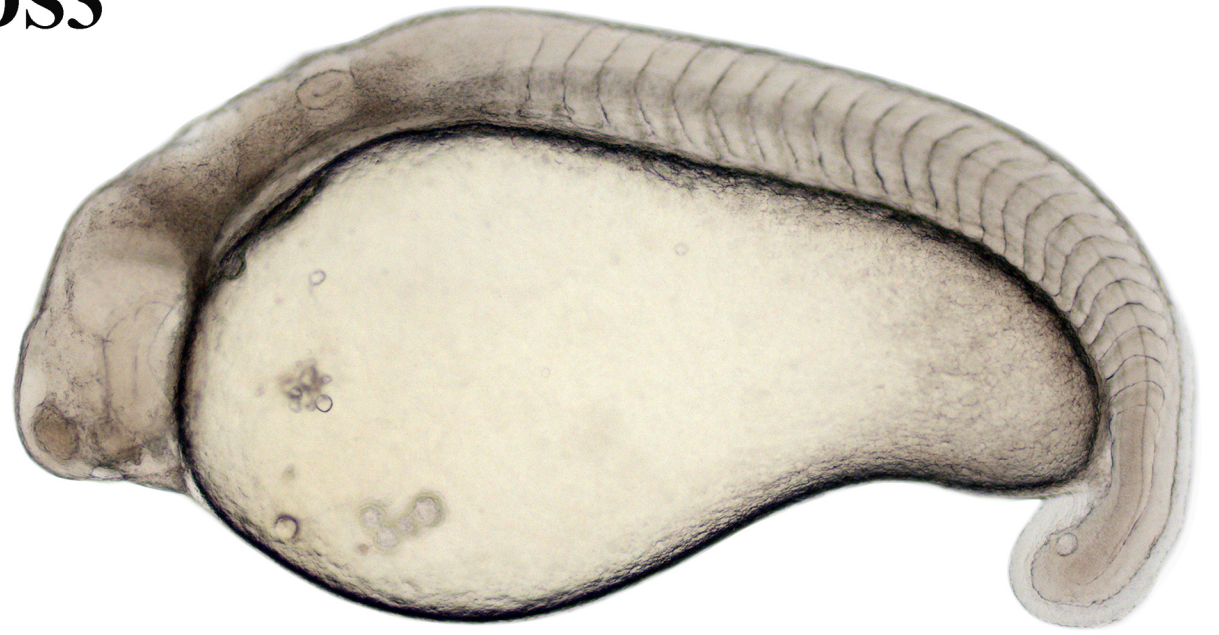

**DS4**

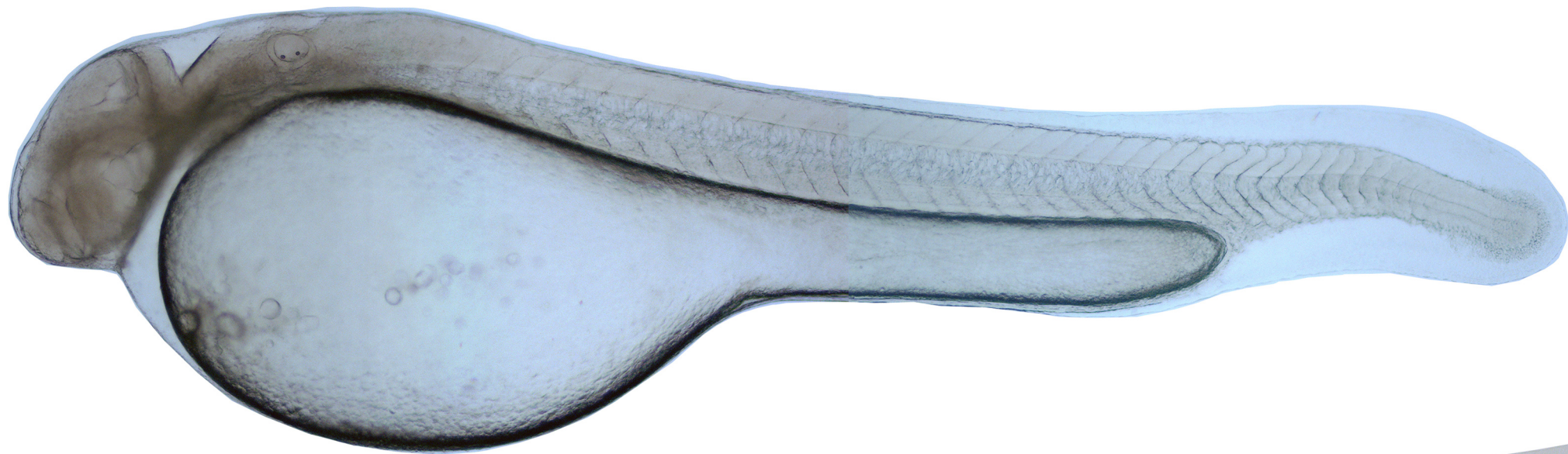

**DS5**

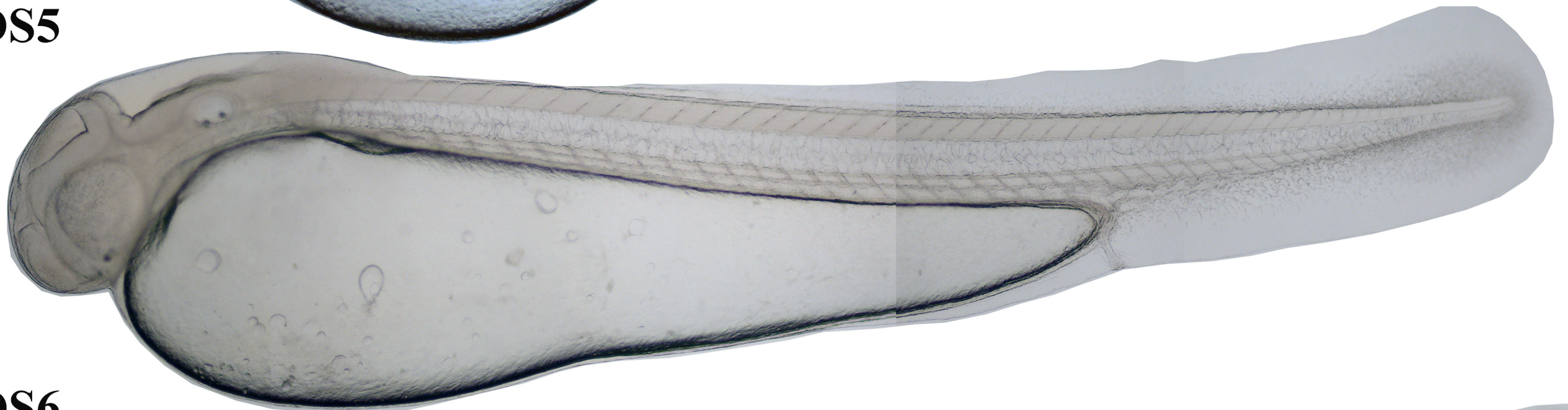

**DS6**

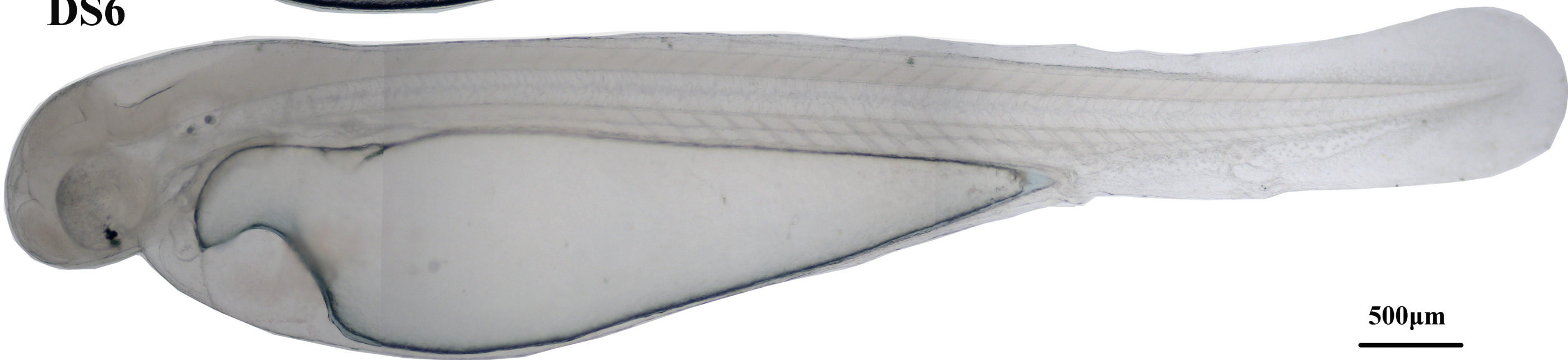

**500μm**

Supplement: Supplementary file 1 — Additional file 1: Figure S1. Six developmental stages sampled in H. nobilis. [file 12864_2019_6181_MOESM1_ESM.pdf]

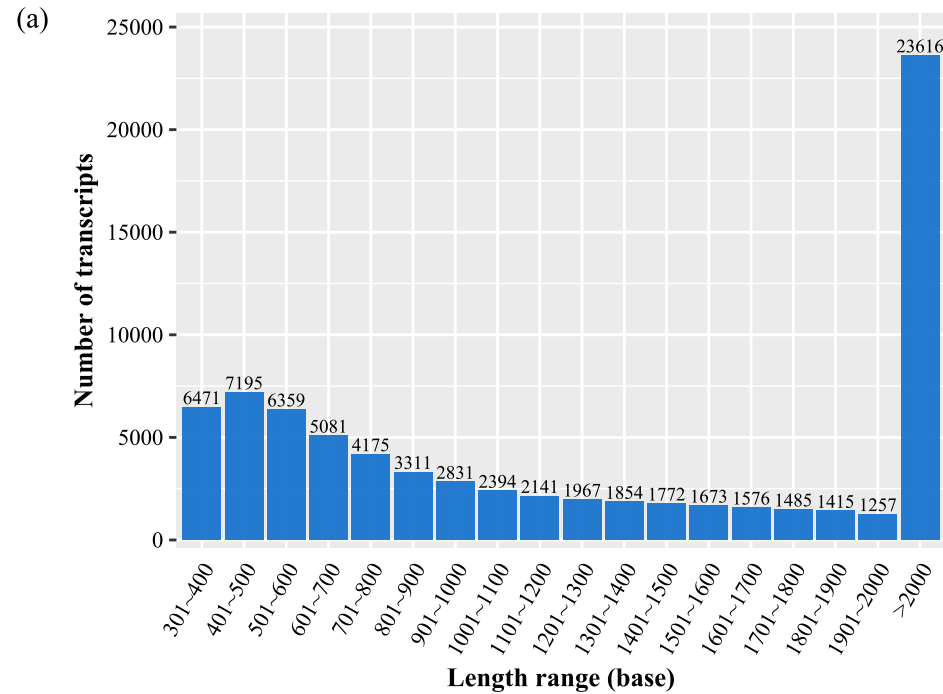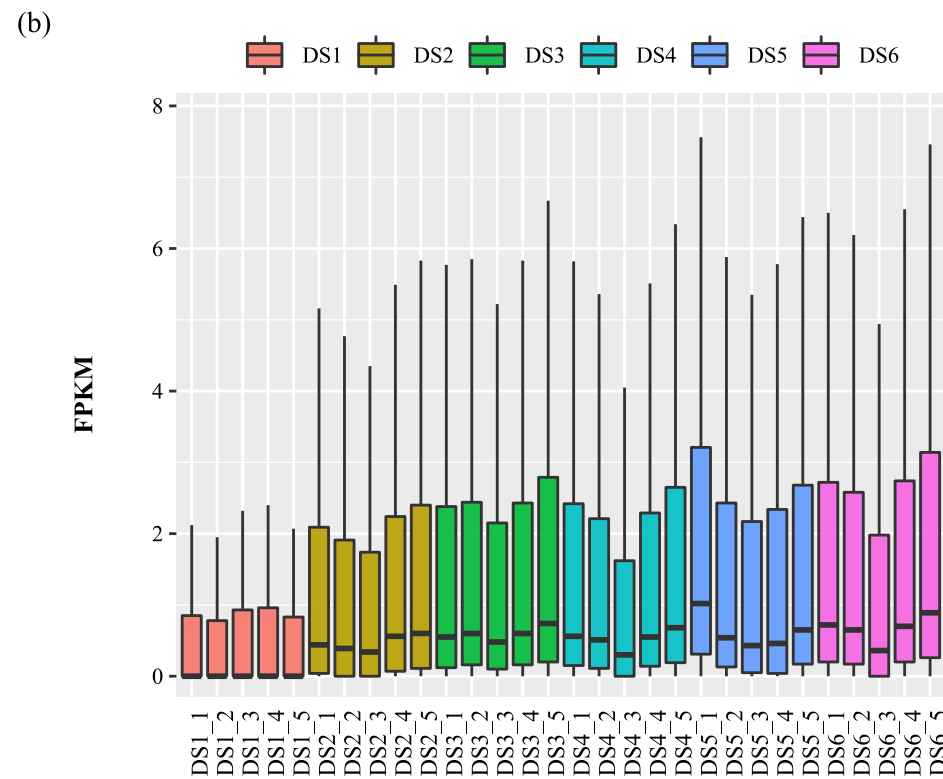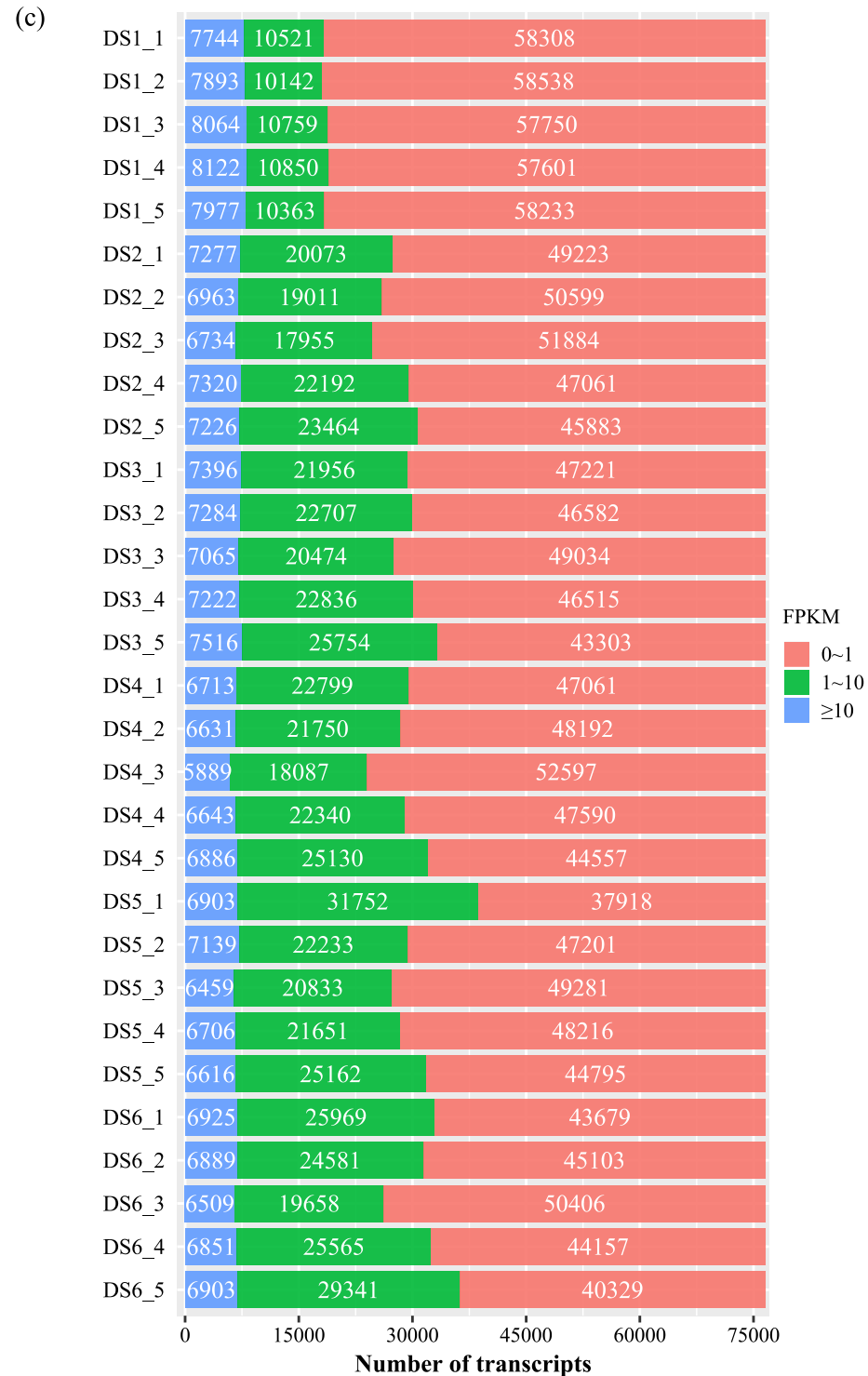

Supplement: Supplementary file 4 — Additional file 4: Figure S2. The length distribution of unigenes assembled in the early developmental stages of H. nobilis (a). The boxplot of Fragments Per Kilobase of transcript per Million mapped reads (FPKM) values (b), and the expression distribution counts in different levels of unigenes in each library (c). [file 12864_2019_6181_MOESM4_ESM.pdf]

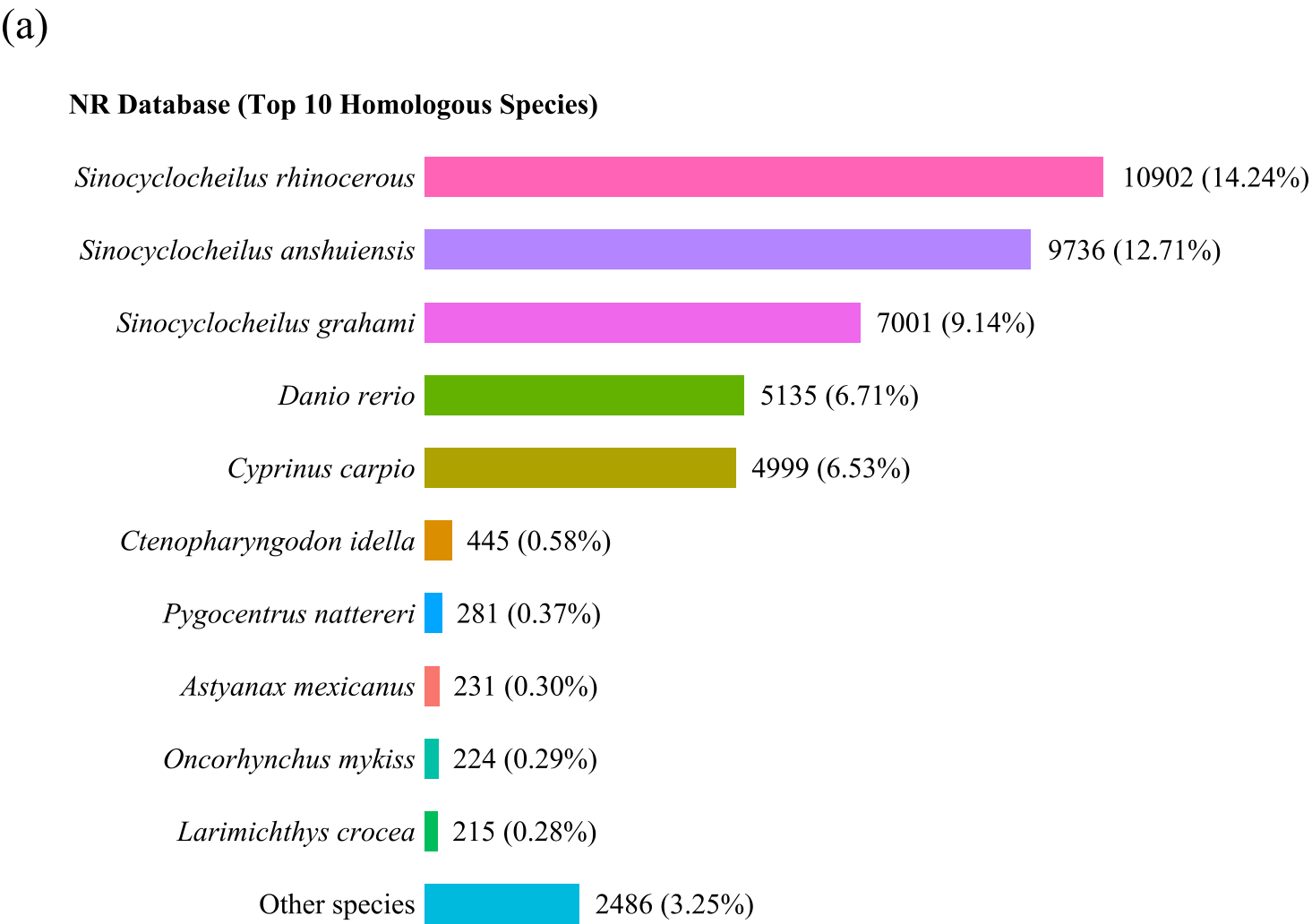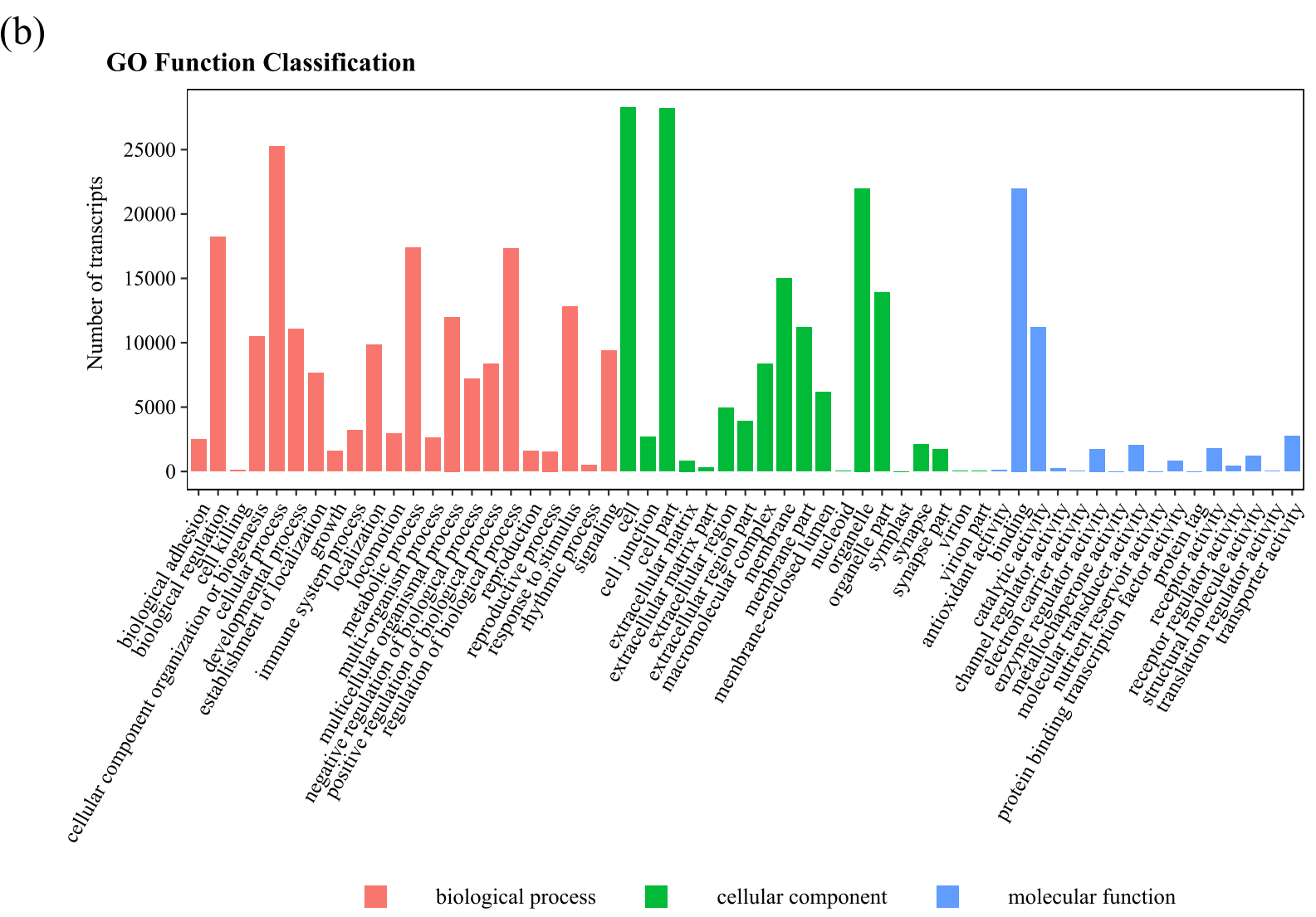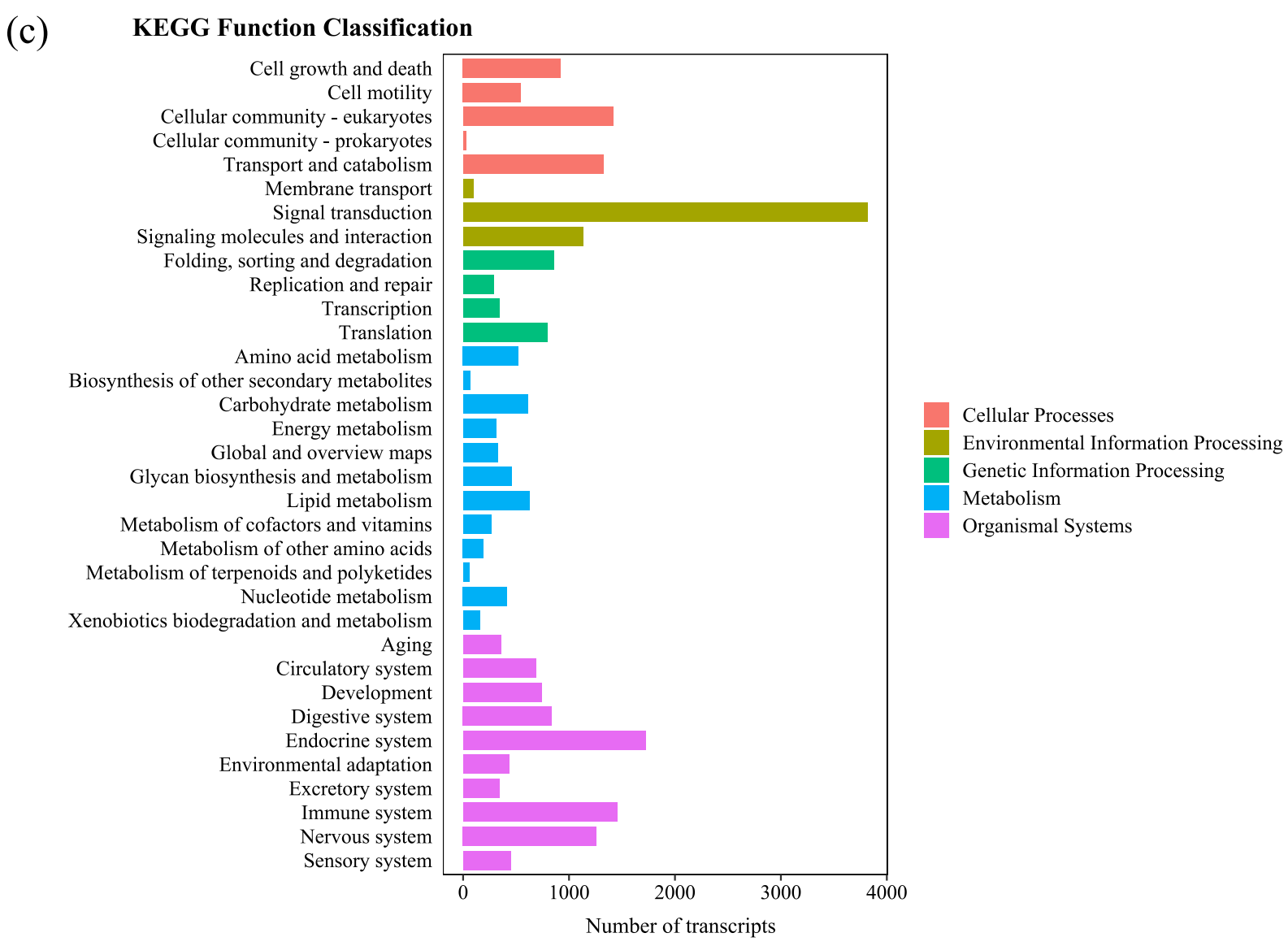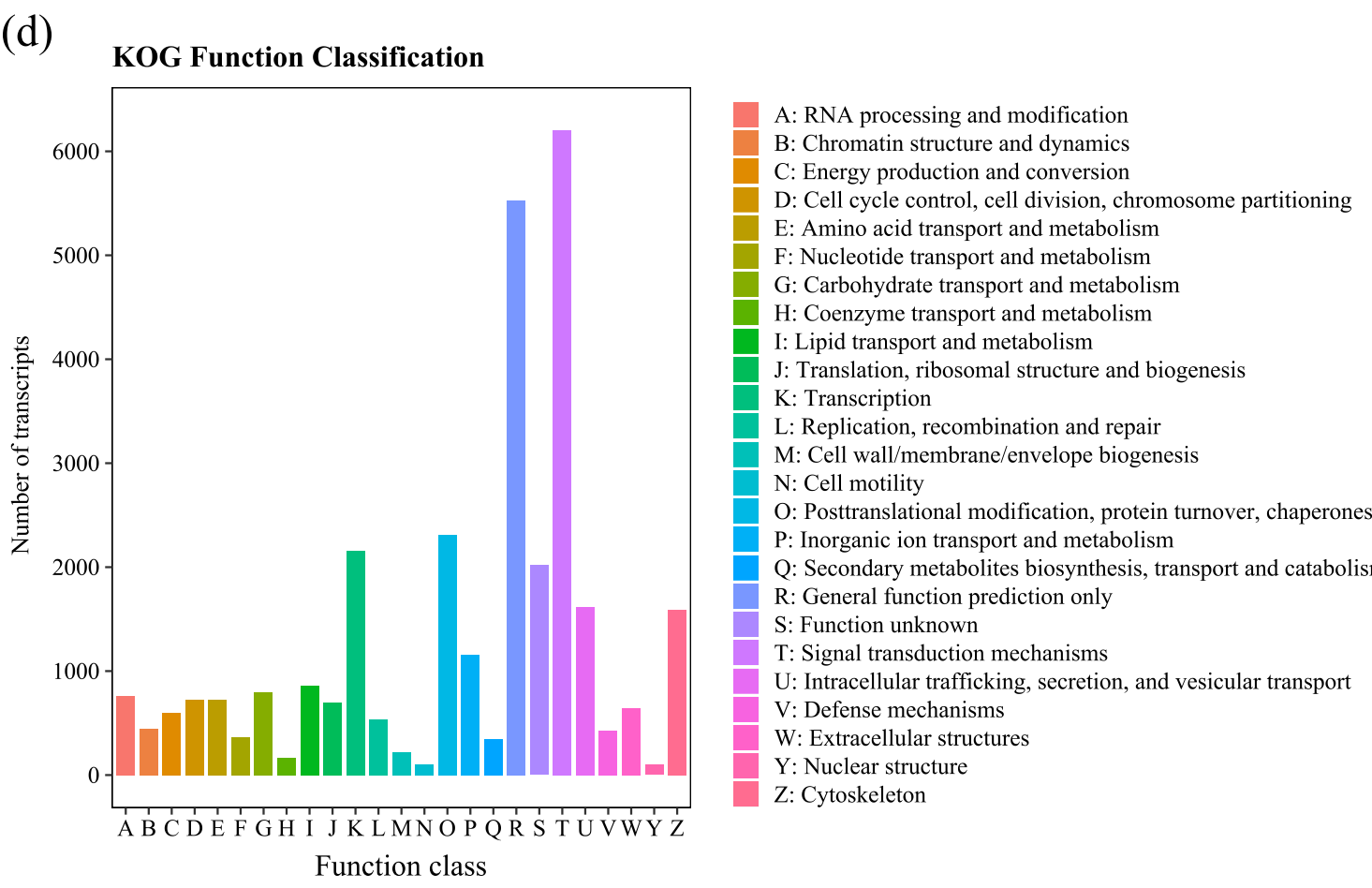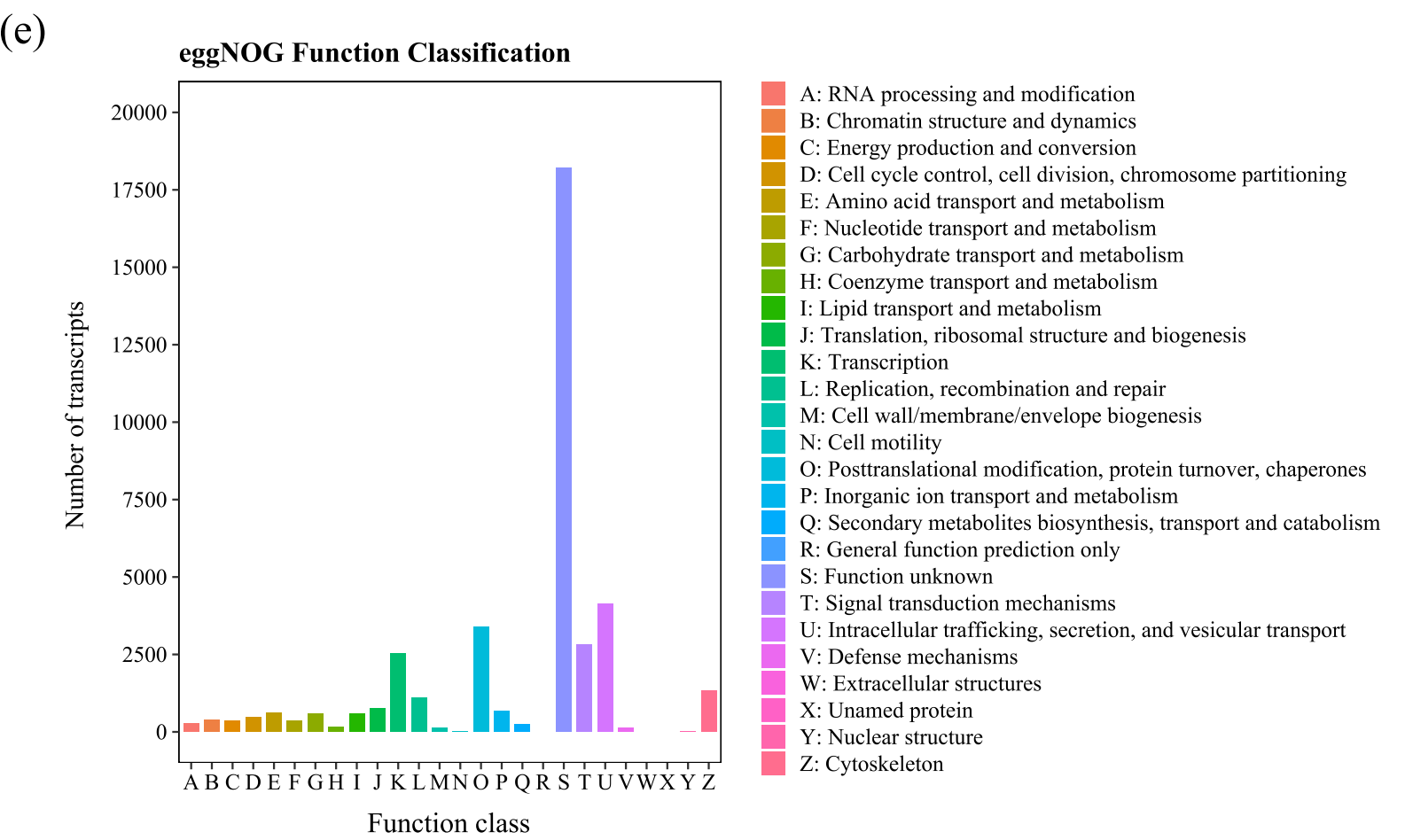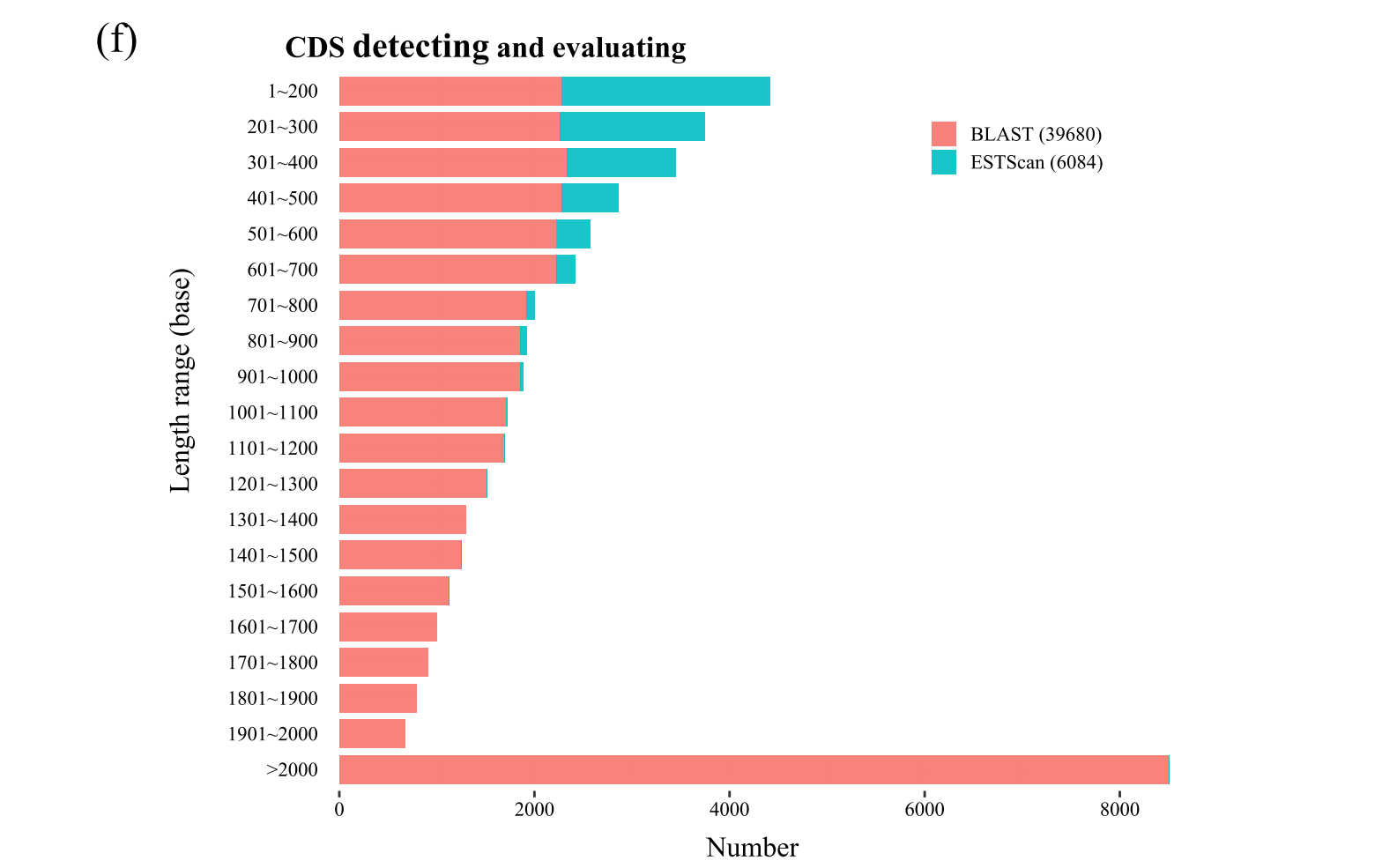

Supplement: Supplementary file 5 — Additional file 5: Figure S3. The annotation statistics of unigenes from five public databases (non-redundant (NR), gene ontology (GO), Kyoto Encyclopedia of Genes and Genomes (KEGG), Eukaryotic orthologous groups (KOG), and evolutionary genealogy of genes: Non-supervised Orthologous Groups (eggNOG), respectively) (a-e), and the coding sequence (CDS) detection and prediction result (f). [file 12864_2019_6181_MOESM5_ESM.pdf]

(a)

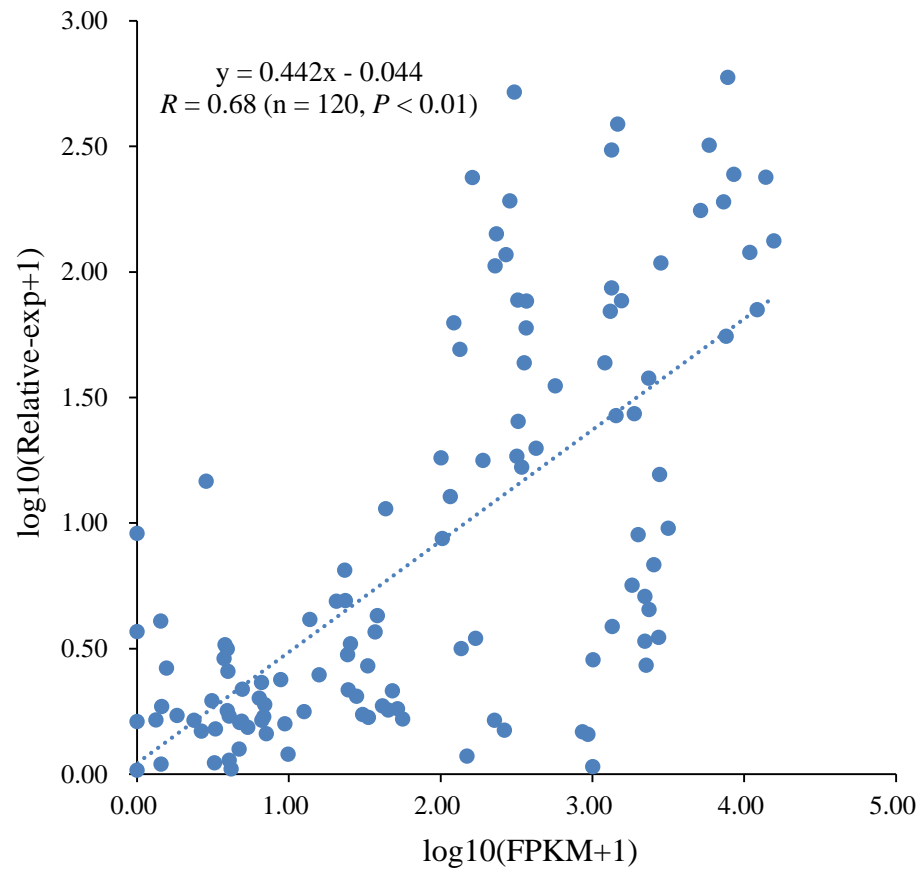

(b)

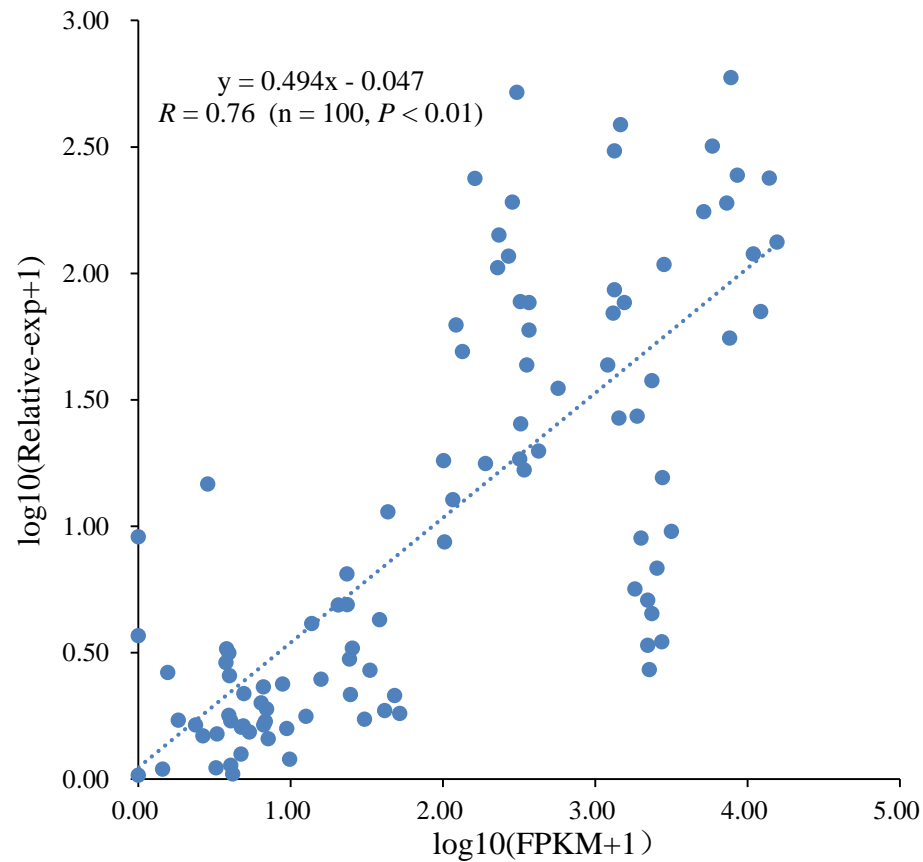

Supplement: Supplementary file 9 — Additional file 9: Figure S4. The correlations of quantitative real-time PCR (qPCR) and Fragments Per Kilobase of transcript per Million mapped reads (FPKM) data of all twelve chosen unigenes (a) and the ten unigenes with coincident expression trend (b). [file 12864_2019_6181_MOESM9_ESM.pdf]

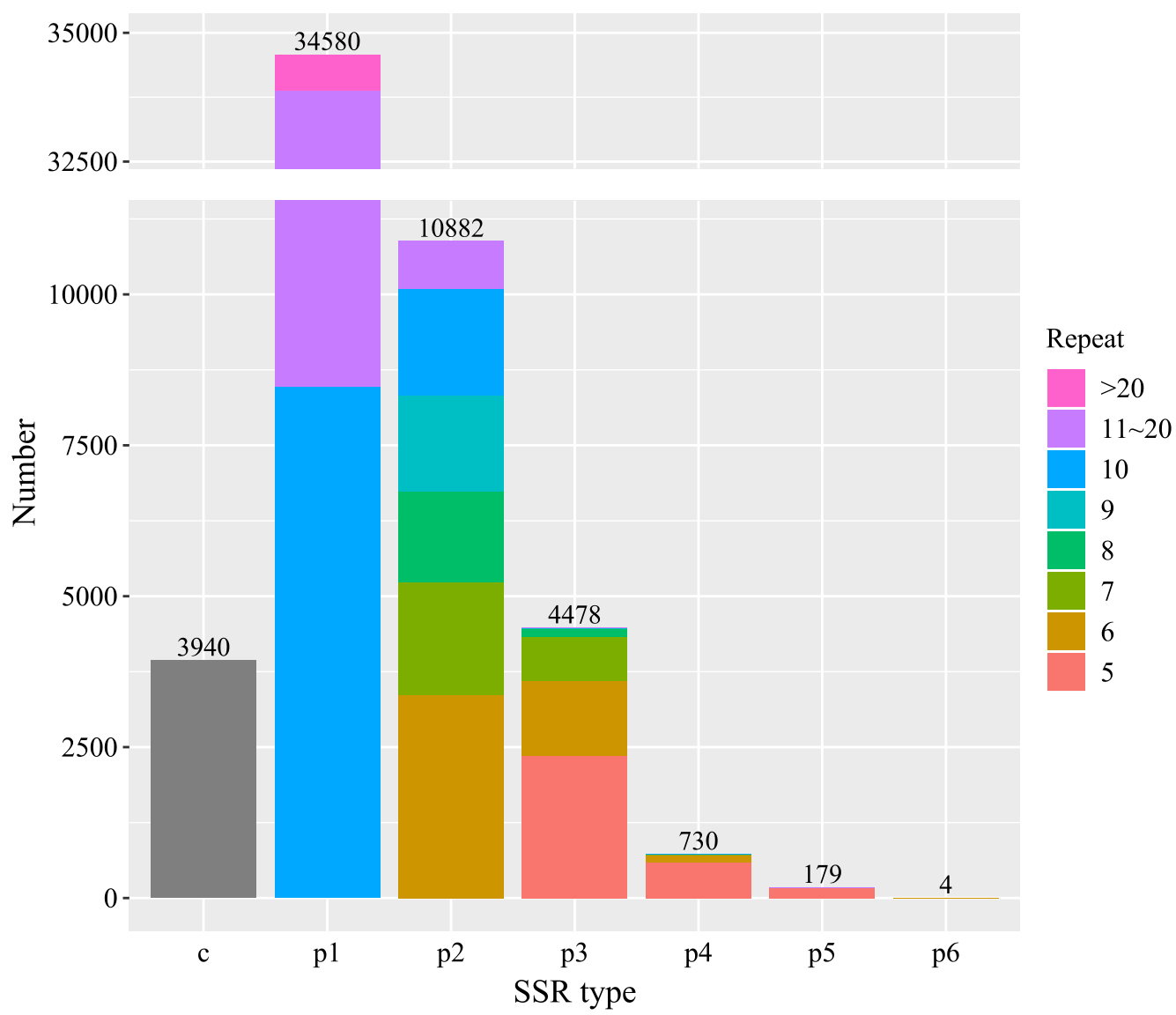

Supplement: Supplementary file 10 — Additional file 10: Figure S5. The statistic of simple sequence repeat (SSR) loci detected in the unigenes from transcriptome sequencing. [file 12864_2019_6181_MOESM10_ESM.pdf]
