# Supplementary material for: Dynamic transcriptome sequencing and analysis during early development in the bighead carp (Hypophthalmichthys nobilis)
Source: BMC Genomics. 2019 Oct 28;20:781. doi: 10.1186/s12864-019-6181-4 (PMC6819325; doi:10.1186/s12864-019-6181-4)
Supplement: Supplementary file 3 — Additional file 3: Table S2. The summary information of de novo assembling. [file 12864_2019_6181_MOESM3_ESM.docx]

**Table S2** The summary information of *de novo* assembling.

| **Terms** | **Value** |
| --- | --- |
| Total (>300base) | 76,573 |
| ≥ 500base | 62,973 |
| ≥ 1000base | 41,172 |
| N50 /base | 2,834 |
| Total length /base | 135,360,228 |
| Max length /base | 32,074 |
| Min length /base | 301 |
| Average length /base | 1,768 |
